# *Chlamydia trachomatis* Infections: Implications for Pregnant Adolescents and Their Infants

**DOI:** 10.1155/S1064744994000323

**Published:** 1994

**Authors:** Marlene Melzer-Lange, Laurie Good, Halim Hennes

**Affiliations:** 1 Department of Pediatrics Medical College of Wisconsin Milwaukee WI USA; 2 Teen Pregnancy Service of Milwaukee Milwaukee WI USA; 3 Department of Pediatrics Children's Hospital of Wisconsin 9000 West Wisconsin Avenue, MS #677 Milwaukee WI 53226 USA

## Abstract

*Objective: Chlamydia trachomatis*  infections are common in pregnant adolescents. Previous studies have shown that treating pregnant women of all ages with erythromycin prevents transmission of this infection to their infants. However, there are no published studies on the efficacy of aggressive screening and treatment of *C. trachomatis* in pregnant adolescents. This study was undertaken to determine if aggressive screening for *C. trachomatis* in pregnant adolescents and early treatment with erythromycin can prevent complications in their newborn infants.

*Methods:* A group of pregnant adolescents enrolled at Teen Pregnancy Service of Milwaukee was evaluated prospectively for the presence of *C. trachomatis* infection. Screening was performed during the 1st and 3rd trimesters by enzyme immunoassay. Adolescents with positive enzyme immunoassays for  *Chlamydia* were treated with erythromycin for 10 days. Those with negative enzyme immunoassays were enrolled as controls. All infants born to adolescents in both groups were followed for episodes of conjunctivitis, pneumonia, and wheezing during their 1st year of life.

*Results:* Ninety mother/infant pairs were followed during the study period. Twenty-eight mothers (31%) had positive enzyme assay tests and all received erythromycin therapy. Nasopharyngeal cultures were obtained from 60 (67%) infants; all were negative. There were no significant differences in general characteristics, development of conjunctivitis (relative risk 1.27), wheezing (relative risk 0.91), or pneumonia (relative risk 1.12) between infants born to  adolescents in either group.

*Conclusions:* We conclude that aggressive screening and treatment of *C. trachomatis* infection in pregnant adolescents may prevent complications in their offspring.


Infectious Diseases in Obstetrics and Gynecology 2:10–15 (1994)
© 1994 Wiley-Liss, Inc.

### *Chlamydia trachomatis* Infections: Implications for Pregnant Adolescents and Their Infants

**Marlene Melzer-Lange, Laurie Good, and Halim Hennes**

*Department of Pediatrics, Medical College of Wisconsin (M.M.-L., H.H.), and Teen Pregnancy Service of Milwaukee (L.G.), Milwaukee, WI*

Abstract

***Objective:*** **Chlamydia trachomatis* infections are common in pregnant adolescents. Previous studies have shown that treating pregnant women of all ages with erythromycin prevents transmission of this infection to their infants. However, there are no published studies on the efficacy of aggressive screening and treatment of *C. trachomatis* in pregnant adolescents. This study was undertaken to determine if aggressive screening for *C. trachomatis* in pregnant adolescents and early treatment with erythromycin can prevent complications in their newborn infants.**

***Methods:*** **A group of pregnant adolescents enrolled at Teen Pregnancy Service of Milwaukee was evaluated prospectively for the presence of *C. trachomatis* infection. Screening was performed during the 1st and 3rd trimesters by enzyme immunoassay. Adolescents with positive enzyme immunoassays for *Chlamydia* were treated with erythromycin for 10 days. Those with negative enzyme immunoassays were enrolled as controls. All infants born to adolescents in both groups were followed for episodes of conjunctivitis, pneumonia, and wheezing during their 1st year of life.**

***Results:*** **Ninety mother/infant pairs were followed during the study period. Twenty-eight mothers (31%) had positive enzyme assay tests and all received erythromycin therapy. Nasopharyngeal cultures were obtained from 60 (67%) infants; all were negative. There were no significant differences in general characteristics, development of conjunctivitis (relative risk 1.27), wheezing (relative risk 0.91), or pneumonia (relative risk 1.12) between infants born to adolescents in either group.**

***Conclusions:*** **We conclude that aggressive screening and treatment of *C. trachomatis* infection in pregnant adolescents may prevent complications in their offspring.** © 1994 Wiley-Liss, Inc.

Key words

Adolescent pregnancy, sexually transmitted disease, prenatal care

*Chlamydia trachomatis* is a common sexually transmitted infection among pregnant adolescents; the infection rate ranges from 14 to 27%.[1–3] Infections during pregnancy are often transmitted to the infant at the time of delivery.[4–6] Rates of transmission vary from 50 to 75% of infants born to infected mothers. Previous studies in women of all childbearing ages have shown that the use of erythromycin during pregnancy diminishes *C. trachomatis* transmission to the infant and prevents complications such as conjunctivitis and pneumonia.[7,8] Adolescents are particularly at risk for poor compliance with prenatal care and medication administration and for reinfection with *C. trachomatis*.[9–11] There are no studies that have examined the effectiveness of erythromycin during pregnancy in a purely adolescent population.

This prospective cohort study was undertaken to

**Address correspondence/reprint requests to Dr. Marlene Melzer-Lange, Department of Pediatrics, Children's Hospital of Wisconsin, 9000 West Wisconsin Avenue, MS #677, Milwaukee, WI 53226.**

**Presented in part at the 32nd Annual Meeting of the Ambulatory Pediatric Association, Baltimore, MD, May 1992.**

Received November 24, 1993

Accepted May 10, 1994

Clinical Study evaluate whether infants born to adolescents treated for *C. trachomatis* with erythromycin during pregnancy were at increased risk for the complications of wheezing, conjunctivitis, and pneumonia compared with infants born to mothers who were negative for *C. trachomatis* during pregnancy.

### SUBJECTS AND METHODS

During a 12-month period, all pregnant adolescents presenting for prenatal care at Teen Pregnancy Service of Milwaukee were screened for *C. trachomatis* infection. An enzyme immunoassay test (Chlamydiazyme™, Abbott Laboratories, North Chicago, IL) was performed at intake, 36 weeks gestation, and 6–8 weeks postpartum. A mother was considered *C. trachomatis* positive if a chlamydial enzyme immunoassay was positive during pregnancy. Adolescents with *C. trachomatis* were treated within 1 week of testing with erythromycin base, 500 mg t.i.d. for 10 days, and retested within 1 month. *C. trachomatis* infections detected at any time during pregnancy were also treated with erythromycin at the same dose. Prenatal testing, treatment, and instruction regarding chlamydial infection were provided to all patients by a certified nurse-midwife on a one-to-one basis. A multidisciplinary program supported all pregnant teens throughout pregnancy with prenatal education classes, social worker intervention, nutritional counseling through the Women Infant Children Nutrition Site (WIC), and group counseling sessions at the same site. Sexual partners were referred for treatment. At the time of delivery, all infants received erythromycin ophthalmic ointment prophylaxis. Demographic data and smoking history were obtained from each patient.

Infants of both *C. trachomatis*-positive and -negative mothers were followed prospectively for 1 year for episodes of conjunctivitis, pneumonia, and wheezing. Visits were scheduled monthly for the 1st 6 months of the infant's life and then every other month until the 1st birthday. Conjunctivitis was defined as erythema with drainage of 1 or both eyes. Pneumonia was defined as rales in conjunction with abnormal chest X-ray. Wheezing was defined as tachypnea associated with expiratory wheezes on auscultation. Episodes were identified during either well-baby visits or sick visits at the clinic or emergency department; history of eye drainage or respiratory distress was requested from care givers at each clinic visit. Access to all medical records for visits was assured since patients were enrolled in a managed-care plan. Nasopharyngeal swabs of infants in both groups were collected between 1 and 6 months of age on Chlamydia Transwab® (Microdiagnostics Corp., Cleveland, OH) and sent directly to the laboratory for McCoy cell culture during well-child visits. Infants with pneumonia or conjunctivitis were also cultured for *C. trachomatis* at the time of their acute illness. Data were analyzed using the Student's t-test, chi-squared test, and Mann-Whitney rank test. Relative risks, with 95% confidence interval, were calculated for outcome variables.

**TABLE 1. General characteristics**

| | *C. trachomatis* positive (N = 28) | *C. trachomatis* negative (N = 62) | *P* |
|---|---|---|---|
| Race | | | |
| Black | 23 (82%) | 46 (74%) | |
| White | 3 (11%) | 6 (10%) | |
| Other | 2 (7%) | 10 (16%) | |
| Maternal age (years)[a] | 16.8 ± 0.3 | 17.3 ± 0.2 | 0.2[c] |
| Infant weight (g)[a] | 3,149 ± 114 | 3,194 ± 75 | 0.7[c] |
| No. of smokers[b] | 8 | 17 | 0.9[d] |
| No. of clinic visits | 10 | 11 | 0.3[c] |

[a]Mean ± SEM.
[b]Total number of smokers in each group.
[c]Student's t-test.
[d]Chi-squared test.

### RESULTS

One hundred ten mother/infant pairs were enrolled and followed during the study period. Twenty pairs were excluded from the study due to change in care providers and loss of follow-up of the infants. There were no differences in the excluded group and the group studied by race, smoking, age, birth weight, or *C. trachomatis* status. Twenty-eight mothers were *C. trachomatis* positive and 62 were *C. trachomatis* negative. Estimated gestational age at intake was 17.2 weeks (range 10–27 weeks) for *C. trachomatis*-positive mothers and 16.9 weeks (range 11–30 weeks) for *C. trachomatis*-negative mothers. The demographic characteristics for the 2 groups are summarized in Table 1. There were no significant differences for maternal age, race, birth weight, or history of maternal smoking. Eighty-eight of 90 (98%) mothers were eligible for Medicaid.

**TABLE 2. Mothers with evidence of *C. trachomatis* during pregnancy[a]**

| Intake | 20 weeks | 24 weeks | 30 weeks | 36 weeks | Postpartum |
|---|---|---|---|---|---|
| (+) N = 22 | | | | (−) N = 18 | (−) N = 18 |
| | | | | (+) N = 2 | (−) N = 2 |
| | (+) N = 1 | | | (−) N = 1 | (−) N = 1 |
| | | | | (−) N = 1 | (+) N = 1 |
| (−) N = 6 | | | | (+) N = 2 | (−) N = 2 |
| | (+) N = 1 | (+) N = 1 | (+) N = 1 | (−) N = 1 | (−) N = 1 |
| | | | | (+) N = 3 | (−) N = 3 |

[a]+ = positive chlamydial enzyme immunoassay.

Chlamydial enzyme immunoassay was positive in 22 mothers at intake. Six additional mothers became positive for *C. trachomatis* during pregnancy. Table 2 summarizes the cervical chlamydial enzyme immunoassay results throughout pregnancy and postpartum.

Of the 28 infants born to mothers treated for *C. trachomatis*, 21 (75%) were cultured for *C. trachomatis*; all were negative. Forty (64%) infants born to *C. trachomatis*-negative mothers were cultured; all were culture negative. There were no positive cultures for *C. trachomatis* during episodes of conjunctivitis or pneumonia.

Of the 28 infants born to mothers treated for *C. trachomatis*, 12 developed conjunctivitis, 2 developed pneumonia, and 12 developed wheezing. Of the 62 infants born to *C. trachomatis*-negative mothers, 23 developed conjunctivitis, 6 developed pneumonia, and 28 developed wheezing. There was no statistical difference between the 2 groups in the number of episodes of upper respiratory infection, conjunctivitis, pneumonia, or wheezing by the Mann-Whitney rank test. Relative risk for conjunctivitis was 1.27, for pneumonia was 1.12, and for wheezing was 0.91 between the 2 groups (Table 3).

### DISCUSSION

The literature provides little data on the compliance of treatment of *C. trachomatis* among pregnant adolescents and the outcome of their infants. Although compliance may be a problem for adolescents, we found that most mothers treated early in their pregnancy for *C. trachomatis* remained disease-free throughout pregnancy. Infants born to mothers treated for *C. trachomatis* showed no evidence of nasopharyngeal carriage nor did they have conjunctivitis, pneumonia, or wheezing more frequently than infants born to *C. trachomatis*-negative mothers.

Adolescents, especially during pregnancy, are at greater risk for *C. trachomatis* infections than adult women. In our study, 31% of pregnant adolescents had *C. trachomatis* infection. Earlier studies[1–3] have reported an infection rate between 14 and 27%. In another study conducted in Milwaukee, 11% of pregnant adult women (mean age 24 years) were found to be infected with *C. trachomatis*.[12] Factors

**TABLE 3. Relationship between disease outcome and maternal *C. trachomatis* results**

| | *C. trachomatis* positive (N = 28) | *C. trachomatis* negative (N = 62) | Relative risk | 95% confidence interval |
|---|---|---|---|---|
| Conjunctivitis | 12 | 23 | 1.27 | 0.52,3.06 |
| No conjunctivitis | 16 | 39 | | |
| Wheezing | 12 | 28 | 0.91 | 0.36,2.21 |
| No wheezing | 16 | 34 | | |
| Pneumonia | 2 | 6 | 1.12 | 0.19,6.4 |
| No pneumonia | 26 | 56 | | |

which account for the high prevalence of *C. trachomatis* in adolescents may include increased sexual activity at an earlier age and multiple sexual partners. Furthermore, pregnant adolescents may also be more likely than nonpregnant adolescents to be infected with *C. trachomatis*. In Brooklyn, NY, the rates were 16% in pregnant adolescents compared with 7.3% in nonpregnant adolescents.[1] Chacko and Lovchik[3] reported *C. trachomatis* rates of 27% in pregnant teens and 23% in nonpregnant teens.

The enzyme immunoassay method was used for the detection of *C. trachomatis* in the endocervical samples from the pregnant adolescents in this study. This method was selected because it is easy to transport and the results can be obtained within 1 day. Though culture by McCoy cell has been the gold standard for detection of chlamydial infection, the enzyme immunoassay has been shown to be appropriate for the screening of pregnant women and adolescents. Baselski et al.[13] showed the enzyme immunoassay (Chlamydiazyme™) to have a sensitivity of 96.3 and a specificity of 92.9% compared with culture in a group of pregnant women. In a study of female adolescents, the enzyme immunoassay demonstrated a sensitivity of 100% and a specificity of 88%.[14]

The timing of *C. trachomatis* screening and erythromycin therapy during pregnancy is controversial. Schachter et al.[7] treated pregnant patients at 36 weeks gestation in hopes of allowing a *C. trachomatis*-free genital tract at the time of delivery. Eight of 152 infants in that study were born while the mother was still taking erythromycin; 2 infants developed *C. trachomatis* infections. Other studies have examined the efficacy of erythromycin for treatment of *C. trachomatis* during pregnancy.[15] However, the efficacy has not been examined in a purely adolescent population.

Compliance, risk-taking behavior, multiple sexual partners, and poor condom use are all factors that may make *C. trachomatis* treatment in pregnant adolescents problematic. We felt that an aggressive approach to the eradication of *C. trachomatis* during the entire gestation of an adolescent pregnancy is appropriate. In our study, pregnant adolescents were screened and treated at intake and again at 36 weeks gestation. Those adolescents who were positive for *C. trachomatis* at intake were aggressively treated and tests of cure were performed 2 weeks after completion of therapy. Although the use of erythromycin during pregnancy has been reported to be associated with poor compliance due to gastrointestinal side effects,[16] the adolescents in our study reported completion of their erythromycin course on their follow-up visits. Early testing of pregnant teens permitted identification and treatment of sexual partners. Any adolescent who reported exposure during pregnancy to *C. trachomatis* from an infected partner was also retested and treated. In the present study, 82% of mothers treated for *C. trachomatis* during the early months of pregnancy remained negative throughout the pregnancy. With aggressive management, *C. trachomatis* was eradicated in an additional 14% of teens prior to delivery. Treatment initiated at detection of *C. trachomatis* at intake was successful in our study and most adolescents remained negative throughout their pregnancy. Patients negative at intake for *C. trachomatis* who became infected later in pregnancy were detected at testing at 36 weeks.

Although current Centers for Disease Control[17] recommendations advise testing and treatment at 36 weeks, our data suggest that evaluation should be done at both the prenatal intake and the 3rd trimester to control chlamydial infection in adolescents.

Another argument may be made to support the recommendation for chlamydial detection early in pregnancy. Recent studies have examined the relationship between *C. trachomatis* infection and premature labor and low birth weight.[15,18] Ryan et al.[19] showed birth weights of <2,500 g in 19% of infants of untreated *C. trachomatis*-positive mothers vs. 11% of treated mothers. Others,[20] however, suspect that mucopurulent cervicitis, unrelated to *C. trachomatis* infection, may be a predictor of adverse pregnancy outcome. Erythromycin therapy, unrelated to *C. trachomatis* infection, may also improve birth weights; McCormack et al.[21] showed fewer low-birth-weight infants in a group of women infected with *Ureaplasma urealyticum* treated with erythromycin during the 3rd trimester. In the present study, birth weights were not significantly different in the 2 groups of patients.

The compliance of the pregnant teens in our study was very good, with most presenting for care by 20 weeks gestation and most receiving 10–11 prenatal visits. Our program offers a multidisciplinary approach to teen pregnancy which includes medical, nutritional, prenatal education, pediatric (for siblings), and social services to the pregnant teen and her children at one inner-city site. Most patients are referred by other adolescents and may partly account for the initiation of care prior to the 3rd trimester. We believe that the compliance of our pregnant adolescents may be better than what could be attained through prenatal care at a site not dedicated to the pregnant teen.

All cultures of infants for *C. trachomatis* were negative. Cell culture by McCoy cell technique was employed for detection of *C. trachomatis* in the infants since nonculture techniques have not been well studied in the infant population. Although no cultures in the study infants were positive for *C. trachomatis*, cultures obtained in the same manner were positive in another group of infants during the same time period.

Because cultures may be somewhat fastidious, infants were followed for symptoms that could be related to *C. trachomatis* infection.[13] In examining conjunctivitis, pneumonia, and wheezing in the 2 groups, we found no differences in risk between infants born to mothers who were *C. trachomatis* negative and those born to mothers treated during pregnancy for *C. trachomatis*. Although *C. trachomatis* may be implicated in 1/3 of infants with pneumonia,[22] we found no increase in risk for pneumonia between the infants born to infected and noninfected mothers. Although *C. trachomatis* conjunctivitis has been found as often as 51% of the time in young infants with conjunctivitis,[23] we found no increase in risk for conjunctivitis between infants born to infected and noninfected mothers.

Wheezing following *C. trachomatis* infection has been reported by several authors.[24–26] Chronic *C. trachomatis* infections in infants have also been reported, some lasting over 2 years.[27] No evidence of increased wheezing or chronic infection was found in infants in the group born to mothers treated for *C. trachomatis*.

The cost of screening and treatment ($84.00) of pregnant teens is far less than the cost of outpatient and in some cases inpatient care of infants with *C. trachomatis* pneumonia. If, as suspected, *C. trachomatis* contributes to long-term chronic respiratory disease in older children, further costs will ensue.[28]

### CONCLUSIONS

Our study demonstrates that screening and treatment for *C. trachomatis* in pregnant adolescents early and throughout pregnancy may be effective in eradicating infection and preventing infectious complications in their offspring. Further studies are needed to investigate the impact that *C. trachomatis* and erythromycin have on the incidence of low birth weight in adolescent pregnancies. Should the newer one-dose antibiotics such as azithromycin be found to be safe for use during pregnancy, treatment may become less complicated. Programs serving pregnant teens must target *C. trachomatis* as a common infection and offer early and persistent evaluation and treatment.

### ACKNOWLEDGMENTS

We thank Margo Kinservik, P.N.P., and Carmen Otero, P.N.P., for their assistance in obtaining infant cultures and Frances Sommer for assistance in preparation of the manuscript. This project was supported in part by a grant from the Wisconsin Perinatal Foundation.

### REFERENCES

1. Golden N, Hammerschlag M, Neuhoff S, Gleyzer A: Prevalence of *Chlamydia trachomatis* cervical infection in female adolescents. Am J Dis Child 138:562–564, 1984.
2. Hammerschlag MR, Cummings C, Roblin PM, Williams TH, Delke I: Efficacy of neonatal ocular prophylaxis for the prevention of chlamydial and gonococcal conjunctivitis. N Engl J Med 320:769–772, 1989.
3. Chacko MR, Lovchik JC: *Chlamydia trachomatis* infection in sexually active adlescents: Prevalence and risk factors. Pediatrics 73:836–840, 1984.
4. Frommell GT, Rothenberg R, Wang SP, McIntosh K: *Chlamydia* infection of mothers and infants. Pediatrics 95:28–32, 1979.
5. Hammerschlag MR, Anderka M, Semine DZ, McComb D, McCormack WM: Prospective study of maternal and infantile infection with *Chlamydia trachomatis*. Pediatrics 64:142–147, 1979.
6. Schachter J, Grossman M, Sweet RL, Holt J, Jordan C, Bishop E: Prospective study of perinatal transmission of *Chlamydia trachomatis*. JAMA 255:3374–3377, 1986.
7. Schachter J, Sweet RL, Grossman M, Landers D, Robbie M, Bishop E: Experience with the routine use of erythromycin for chlamydial infections in pregnancy. N Engl J Med 314:276–279, 1986.
8. Heggie AD, Lumicao GG, Stuart LA, Gyves MT: *Chlamydia trachomatis* infection in mothers and infants. Am J Dis Child 135:507–511, 1981.
9. Bell TA, Farrow JA: Compliance with treatment for sexually transmitted diseases. Semin Adol Med 3:153–156, 1987.
10. Smith PB, Chacko MR, McGill L, Phillips LE: Sexually transmitted disease treatment and return for test of cure of adolescents in a family planning clinic. J Adol Health 12:49–52, 1991.
11. Katz BP, Zwickl BW, Caine VA, Jones RB: Compliance with antibiotic therapy for *Chlamydia trachomatis* and *Neisseria gonorrhoeae*. Sex Transm Dis 19:351–354, 1992.
12. Baumgardner DJ, Christopherson A, Momont S: *Chlamydia* in pregnant women: Southwestern Wisconsin. Wis Med J 88(9):12–15, 1989.
13. Baselski VS, McNeeley SG, Ryan G, Robison M: A comparison of nonculture-dependent methods for detection of *Chlamydia trachomatis* infections in pregnant women. Obstet Gynecol 70:47–52, 1987.
14. Krowchuk DP, Anglin TM, Lembo RM, Brown RF, Thomas F, Kumar ML: Use of enzyme immunoassay for the rapid diagnosis of *Chlamydia trachomatis* endocervical infection in female adolescents. J Adol Health Care 9:296–300, 1988.
15. Sweet RL, Landers DV, Walker C, Schachter J: *Chlamydia trachomatis* infection and pregnancy outcome. Am J Obstet Gynecol 156:824–833, 1987.
16. Alger LS, Lovchik JC: Comparative efficacy of clindamycin versus erythromycin in eradication of antenatal *C. trachomatis*. Am J Obstet Gynecol 165:375–381, 1991.
17. Centers for Disease Control: Recommendations for the prevention and management of *Chlamydia trachomatis* infections. MMWR 42:1–39, 1993.
18. Cohen I, Veille JC, Calkins BM: Improved pregnancy outcome following successful treatment of chlamydial infection. JAMA 263:3160–3163, 1990.
19. Ryan GM, Abdella TN, McNeeley SG, Baselski VS, Drummond DE: *Chlamydia trachomatis* infection in pregnancy and effect of treatment on outcome. Am J Obstet Gynecol 162:34–39, 1990.
20. Nugent RP, Hillier SL: Mucopurulent cervicitis as a predictor of chlamydial infection and adverse pregnancy outcome. Sex Transm Dis 19:198–202, 1992.
21. McCormack WM, Rosner B, et al.: Effect on birth weight of erythromycin treatment of pregnant women. Obstet Gynecol 69:202–207, 1987.
22. Brasfield DM, Stagno S, Whitley RJ, Cloud G, Cassell G, Tiller RE: Infant pneumonitis associated with cytomegalovirus, *Chlamydia*, *Pneumocystis*, and *Ureaplasma*: Follow-up. Pediatrics 79:76–83, 1987.
23. Barry WC, Teare EL, Uttley AHC, et al.: *Chlamydia trachomatis* as a cause of neonatal conjunctivitis. Arch Dis Child 61:797–799, 1986.
24. Bavastrelli M, Midulla M, Rossi D, Salzano M: *Chlamydia trachomatis* infection in children with wheezing simulating asthma. Lancet 339:1174, 1992.
25. Carballal G, Mahony JB, Videla C, Cerqueiro C, Chernesky M: chlamydial antibodies in children with lower respiratory disease. Pediatr Infect Dis J 11:68–71, 1991.
26. Weiss SG, Newcomb RW, Beem MO: Pulmonary assessment of children after chlamydial pneumonia of infancy. J Pediatr 108:659–664, 1986.
27. Bell TA, Stamm WE, Wang SP, Kuo CC, Holmes KK, Grayston JT: Chronic *Chlamydia trachomatis* infections in infants. JAMA 267:400–402, 1992.
28. Harrison HR, Phil D, Taussig LM, Fulginiti VA: *Chlamydia trachomatis* and chronic respiratory disease in childhood. Pediatr Infect Dis J 1:29–33, 1982.
